# The Role of Echocardiography and Cardiac Computed Tomography in Diagnosis of Infective Endocarditis

**DOI:** 10.3390/jcm12175482

**Published:** 2023-08-25

**Authors:** Ana Petkovic, Nemanja Menkovic, Olga Petrovic, Ilija Bilbija, Nikola N. Radovanovic, Dejana Stanisavljevic, Svetozar Putnik, Ruzica Maksimovic, Branislava Ivanovic

**Affiliations:** 1 Diagnostic Department of Center of Stereotaxic Radiosurgery, Clinic of Neurosurgery, University Clinical Center of Serbia, 11000 Belgrade, Serbia; ancipetkovic@gmail.com (A.P.); nemanjam59@hotmail.com (N.M.); 2 Cardiology Clinic, University Clinical Center of Serbia, School of Medicine, University of Belgrade, 11000 Belgrade, Serbia; opetrovic1976@gmail.com; 3 Department for Cardiac Surgery, University Clinical Center of Serbia, School of Medicine, University of Belgrade, 11000 Belgrade, Serbia; i.bilbija@yahoo.com (I.B.); svetozar073@yahoo.com (S.P.); 4 Pacemaker Center, University Clinical Center of Serbia, School of Medicine, University of Belgrade, 11000 Belgrade, Serbia; nikolar86@gmail.com; 5 Institute for Medical Statistics and Informatics, Faculty of Medicine, University of Belgrade, 11000 Belgrade, Serbia; sdejana8@yahoo.com; 6 Center of Radiology, University Clinical Center of Serbia, School of Medicine, University of Belgrade, 11000 Belgrade, Serbia

**Keywords:** infective endocarditis, transthoracic echocardiography, transesophageal echocardiography, cardiac computed tomography

## Abstract

Background: Infective endocarditis (IE) is a rare disease with a high mortality rate and rising incidence, requiring timely and precise diagnosis in order to choose appropriate therapy. Imaging of morphologic lesions is an integrative part of diagnosis. Artifacts and the patient’s habitus make echocardiography difficult to visualize advanced-form IE. Cardiac computed tomography (CCT) constantly shows an additive diagnostic value due to high resolution of cardiac anatomy. Conjecturally, joint application of both diagnostic tests improves overall sensitivity and specificity in diagnosing IE. Methods: Patients with definite IE underwent transthoracic echocardiography (TTE), transesophageal echocardiography (TEE), and CCT. We analyzed valvular and paravalvular IE lesions in all three imaging methods and compared them to surgical or autopsy findings. We calculated sensitivity, specificity, diagnostic accuracy, and positive and negative predictive value of both imaging tests individually and jointly used. Results: We examined 78 patients, male to female ratio 2:1, mean age 52.29 ± 16.62. We analyzed 85 valves, 70 native valves, 13 prosthetic valves, and 2 corrected valves due to Ozaki procedure, along with a central shunt and 4 pacemaker leads. As a single test, the sensitivity and specificity of CCT, TTE, and TEE for valvular lesions were 91.6/20%, 65.5/57.9%, and 60/84%, and paravalvular lesions were 100/0%, 46/10.5%, and 14.7/100%. When combined together, sensitivity and specificity for valvular lesions rose to 96.6/0% and paravalvular lesions to 100/0%. We also analyzed the diagnostic performance for each test in single and mutual application, per specific IE lesion. Conclusion: In the individual application, CCT in comparison to TTE and TEE shows better diagnostic performance in detection of valvular and paravalvular lesions. In joint application, there is a statistically significant difference in performance compared to their single use, especially in prosthetic valves and invasive forms of IE native valves.

## 1. Introduction

Infective endocarditis (IE) is a localized infection of the endocardium. It can involve native and prosthetic valves or intracardiac devices [1,2,3].

The disease is accompanied by a high mortality rate despite significant progress in the diagnostic and therapeutic approach [4]. It is considered a rare disease with an increasing incidence [5]. Risk factors have changed toward patients with degenerative heart valves, diabetes, cancer, and congenital heart defects (CHD) [1]. Older population and male persons are affected more often, but it has become common in children with CHD [1,6]. With the development of invasive medical procedures, staphylococcus has become the leading cause of IE in about 30% of cases [1,2,3].

The diagnosis of IE requires the integration of clinical, microbiological, and imaging findings based on the Duke’s criteria [1,7,8,9]. Imaging plays a key role and the mainstay is echocardiography, accompanied with additional imaging modalities, such as cardiac CT, magnetic resonance imaging, and positron emission tomography/computed tomography (PET/CT). Their role in the assessment of IE is threefold: hemodynamic, anatomical, and functional [10].

The main goal of imaging is to confirm the presence of a cardiac lesion, valvular and/or paravalvular [11,12,13]. Echocardiography has a crucial role in assessing the severity of the disease, predicting short-term and long-term outcomes, managing complications, and directing the patient’s therapy [14]. Destruction of the valvular apparatus with the consequent development of ventricular dysfunction and vegetation with embolic potential are decisive in the decision concerning surgical treatment [15]. When TTE is negative and the suspicion of IE is high, TEE is advised to confirm or rule out the diagnosis, but artifacts and patient habitus can make detection of IE lesions difficult [12,16,17].

Taking into account the imperfection of Duke’s criteria and the shortcomings of echocardiography, there is a need for a newer approach in cardiac imaging. CCT consistently shows additive diagnostic value in the follow-up of patients with suspected IE, due to depiction of cardiac anatomy in high-resolution.

Recommended indications for cardiac CT include:

- The presence of anatomical obstacles in the performance of TEE and intolerance or refusal of TEE;
- The presence of metallic material in the heart;
- Initial negative or inconclusive TEE in the case of existing suspicion of IE;
- Suspicion of perivalvular infection;
- Planning cardiac surgery [18,19].

The main hypothesis is that the joint application of imaging modalities, echocardiography, and cardiac computed tomography improves the spatial and temporal resolution of both diagnostic tests, thereby improving the detection of morphological characteristics of infective endocarditis compared to the individual application of both diagnostic tests.

A secondary hypothesis is whether technically advanced CT devices, with voltage and current modulation and an iterative model of reconstructed sinogram data, can reduce radiation exposure when performing cardiac CT, while maintaining the image quality.

## 2. Material and Methods

### 2.1. Study Design and Population

We performed the diagnostic cross-sectional study as approved by the Ethics Committee of the School of Medicine, University of Belgrade. We examined 83 patients who satisfied the category of definite and possible IE, based on the modified Duke’s criteria, according to the recommendations of the European Association of Cardiology from 2015, at the University Clinical Center of Serbia between May 2013 and April 2023. The following patient characteristics were collected: age, gender, heart rate, ejection fraction, hemoculture, causative microorganisms, laboratory finding (leucocyte count, C-reactive protein, fibrinogen, and procalcitonin), and presence of systemic emboli. We observed the presence of risk factors: degenerative valve disease, bicuspid valve, mitral valve prolapse, uncorrected and corrected congenital heart defect, presence of prosthetic valve, and pacemaker. We also observed the presence of coronary artery disease (absent, intermediate, and significant, and presence of mycotic pseudoaneurysms).

The subject of our research was the detection and localization of morphologic features of IE, including valvular and paravalvular lesions, by two diagnostic tests: echocardiography (TTE and TEE) and electrocardiographic (ECG)-gated CCT. The observational categories of valvular lesions were vegetations, aneurysms, and perforations. The observational categories of perivalvular lesions were abscesses, pseudoaneurysms, fistulas, and leak. Features of IE were observed on the native and prosthetic valves and in the implanted cardiac devices. With NVE and PVE, we observed the presence of signs in four potential locations—aortic, mitral, pulmonary, and tricuspid valves. The operative and the autopsy findings of both diagnostic tests were compared with a control group. Seventy-eight patients were enrolled in the study.

### 2.2. Transthoracic and Transesophageal Echocardiography

The patients with suspected IE underwent echocardiographic examinations prior to CT examination according to the standard clinical protocol. The echocardiographic examination always included TTE together with TEE, except in cases where it could not be performed, with a 3 to 5 day interval. Echocardiography was performed by using a General Electric Vivid E95 ultrasound machine; TTE was performed with a cardiac sector probe, M5Sc-D (1.4–4.6 MHz), and TEE was performed with a Volume TEE Probe 6VT-D (3.0–8.0 MHz) transducer.

### 2.3. ECG-Gated Cardiac Computed Tomography

ECG-gated CCT was performed 3 to 7 days after the echocardiographic examination. All CT scans were performed on a 64-row CT system (General Electric VTC Light Speed) and a 64-slice CT system (Siemens Definition AS). After a scout view, unenhanced sequential prospective ECG-gated CT examination was performed, followed by contrast-enhanced helical retrospective ECG-gated CT examination. All CT scans were performed with biventricular contrast injection protocol, followed by saline flush. Dose of contrast medium (CM) was calculated according to body weight, approximately 1.5 mL/kg, and administered intravenously at flow rate 5–7 mL/s, depending on the calculated total amount of CM. In the first phase, we administered 2/3 of the entire CM. In the second phase, we administered the remaining 1/3 of the contrast medium mixed with saline in a proportion dependent on whether the patient had right-sided or left-sided IE. In right-sided IE, the proportion was 50% CM and 50% saline in a slower flow rate (approximately 3 mL/s) in order to avoid turbulent flow in the right atrium and ventricle as much as possible. In left-sided IE, the proportion was 70% in favor of saline. In the third phase, we administered 30 mL pure saline. The time to administer CM, relative to the beginning of the contrast-enhanced helical retrospective ECG-gated examination, was determined with the time bolus technique described previously, with the placement of the region of interest at the aortic root, at the level of the origin of the left main artery. Row data acquired using the 64-row CT system (General Electric VTC Light Speed) were reconstructed with filtered back projection (FBP) at 120 kV tube voltage, without dose modulation of tube current and voltage. Row data acquired using the 64-slice CT system (Siemens Definition AS Serial No. 91985 SW VA48A) were reconstructed with sinogram-affirmed iterative reconstruction (SAFIRE), divided into two groups, one with dose modulation of the tube voltage and current and the other without, with predefined tube voltages at 120 and 140 kV, dependent on body mass index. Retrospective image data were reconstructed at 10–90% of the R-R interval of the cardiac phase and the best diastole phase. The analysis was performed by using Siemens Workstation syngo.via Model VB30B. From multiplanar reformation, we used double-oblique reformation in order to create the aortic, mitral, tricuspid, and pulmonary valve plane, and then we performed analysis in oblique-transversal and oblique-longitudinal sections. Morphologic characteristics of valvular lesions using CCT follow: Vegetation was defined as hypodense lesions attached to the cusps of the valve from the attachment, near commissure, to the free edge, longer than 3 mm. Aneurysm was defined as outpouchings of the valve contour, clearly demarcated from their normal curvature. Perforation was defined as abruption of the valve continuity, clearly demarcated from the valve orifice. Morphologic characteristics of paravalvular lesions using CCT follow: Abscess was defined as thick and hypodense paravalvular lesions, clearly demarcated from the density of surrounded fat. Pseudoaneurysm was defined as a contrast-filled cavity around the valve. Leak was defined as a thin, contrast-filled cavity near the prosthetic valvula, clearly different from the sewing ring. Fistula was defined as a contrast-filled tunnel connecting two neighboring cavities. All lesions were confirmed in two planes. Vegetation length and width were measured using CCT examination, aneurysm depth and width, perforation width, abscess and pseudoaneurysm depth and width, and fistula and leak width.

### 2.4. Technical Criteria for Image Quality Improvement

To determine the image quality assigned by the observer (low, satisfactory, or excellent), we analyzed tube voltage and the type of reconstructions of row data, filter back projection (FBP), or iterative reconstruction.

### 2.5. Statistical Analysis

Continuous variables are expressed as mean ± standard deviation, and nominal variables are presented as percentages. The diagnostic performance (diagnostic accuracy, sensitivity, specificity, and positive predictive and negative predictive value) of the categories for each imaging finding detected on TTE, TEE, and CCT were determined by comparing the results with those of the surgical or autopsy finding (the reference standard). The numbers of true positive, false positive, and false negative cases of TTE, TEE, and CCT for demonstrating each imaging finding of IE are presented. The actual true negative number of each modality could not be derived from the study because of the small number of patients and patients’ selection criteria. In cross-tabulation analysis, we compared the type of reconstructions and tube voltage to the image quality. The hypotheses were tested with Pearson’s chi-squared quadrat test and Student’s *t*-test; a value of *p* < 0.05 was considered significant. The statistical analysis was performed using SSPS version 20.0 (SSPS Inc., Chicago, IL, USA).

## 3. Results

### 3.1. Patients and Valves

Patients’ demographic and clinical data are summarized in Table 1.

Seventy-eight patients had CCT and TTE, whereas twenty patients did not have TEE. We analyzed 85 valves, out of which there were 70 native, 13 prosthetic, and 2 corrected aortic valves due to Ozaki procedure, along with one central shunt and four pacemaker leads. Among 70 native valves, there were 42 aortic, 23 mitral, 3 tricuspid, and 2 pulmonary valves. Among 13 prosthetic valves, there were 11 aortic, 1 mitral, none tricuspid, and 1 pulmonary prosthetic valve. Using CCT, we found 251 morphologic lesions of IE, and 181 valvular and 70 paravalvular lesions. Among valvular lesions, we found 88 vegetations, 42 aneurysms, and 50 perforations. Among paravalvular lesions, we found 20 abscesses, 46 pseudoaneurysms, 2 leaks, and 3 fistulas. Among all 251 lesions, 204 were found on native and 47 on prosthetic valves, shunt, and pacemaker leads.

### 3.2. Diagnostic Performance of Echocardiography and Cardiac Computed Tomography

In detecting all morphological lesions of IE, a single usage of diagnostic tests detected the following lesions: TTE 103 out of 251, TEE 105 out of 198, and CCT 210 out of 251. All three diagnostic tests, in mutual usage, detected 174 lesions out of 198. Consequentially, sensitivity was raised to 97.8%, versus individual application for TTE 46.2%, TEE 58.7%, and CCT 94.2%.

In detection of valvular lesions, TTE detected 93 lesions out of 180, TEE 76 out of 133, and CCT 142 out of 180, but in mutual application, 112 out of 134. Combined usage of all three tests elevated the sensitivity to 96.6%, in contrast to single application for TTE 60.0%, TEE 65.5%, and CCT 91.6%.

In the detection of paravalvular lesions, true positive findings for single applied imaging tests, as opposed to the total number of lesions, were TTE 10 (71), TEE 29 (65), and CCT 68 (71), whereas for TTE + TEE + CCT the result was 62 (64). Additionally, the sensitivity was raised to 100% in their mutual application concerning solitary TTE (14.7%) and TEE (46%), but was the same as in the singularly applied CCT. Performance of diagnostic tests is summarized in Table 2.

### 3.3. Vegetations

Concerning detection of vegetations in all of the valves, tests used individually detected the following number of true positive findings in comparison to total number of vegetations: TTE 70 (88), TEE 48 (65), and CCT 78 (88), whereas mutually applied tests detected 61 vegetations out of 66. The sensitivity and specificity in single usage tests were TTE 84.3%/20%, TEE 78.7%/25%, and CCT 94%/60%. When all three imaging tests were used mutually, the sensitivity rose to 100%, but specificity was 0%.

In the native valves, TTE detected 60 vegetations out of 74, TEE 41 out of 54, and CCT 68 out of 74, but in mutual application, TTE + TEE + CCT detected 50 out of 55. The calculated sensitivity and specificity for diagnosing vegetation at native valves were TTE 87%/20%, TEE 82%/25%, and CCT 98.6%/60%. When combined together, the sensitivity rose to 100%, but specificity dropped to 0%.

In the prosthetic valves only, for tests used individually, TTE detected 10 vegetations out of 14, TEE 7 out of 11, and CCT 10 out of 14. Mutually applied tests detected all 11 vegetations. The sensitivity was TTE 71.4%, TEE 63.6%, and CCT 71.4%. As expected, when used together, the sensitivity rose to 100%.

In CCT examination, the mean length of vegetation was 13.04 ± 6.04 mm (95% confidence interval (Cl) 11.67–14.42), with minimal value 3.30 mm and maximum value 32.80 mm. The mean width of vegetations was 4.55 ± 3.58 mm (95% Cl 3.72–5.38), with minimal value 1 mm and maximum value 26.8 mm.

Performance of the diagnostic tests is summarized in Table 3.

### 3.4. Aneurysms

In single usage of the imaging test, the sensitivity and specificity for detection of aneurysms were TTE 8.7% and 100%, TEE 31.6% and 75.0%, and CCT 100% and 10.5%. In mutual usage of imaging tests, the sensitivity for detection of aneurysms was 100%, as it was in single usage of CCT.

In CCT examination, the mean depth of aneurysms was 7.16 ± 2.65 mm (95% Cl 6.31–8.01) and the minimal to maximal values were 3.7 and 15.2 mm. The mean width of aneurysms was 8.54 ± 4.25 mm (95% Cl 7.19–9.90), with minimal value 3.7 mm and maximal value 19.8 mm.

Performance of diagnostic tests is summarized in Table 4.

### 3.5. Perforations

In the diagnostic performance of tests used individually, the sensitivity and specificity in detection of perforations were TTE 42.9% and 100%, TEE 63.9% and 100%, and CCT 83.7% and 0%. In combined use of the imaging tests, TTE + TEE + CCT, the sensitivity and specificity were 88.9% and 0%.

The width of perforations in the CCT examination was 5.13 ± 2.12 mm (95% Cl 4.45–5.81), with minimal value 1.8 mm and maximal value 10.8 mm.

Performance of diagnostic tests is summarized in Table 5.

### 3.6. Abscesses

Single-applied imaging tests detected the following numbers of abscesses around all types of valves: TTE 3 (out of 20), TEE 8 (out of 19), and CCT 19 (out of 20). When combined together, 18 abscesses (out of 19) were detected. The sensitivity for single-applied TTE was 15.8% and TEE 44.4%, but single-applied CCT and all three tests mutually applied had the exact sensitivity of 100%.

In single usage of imaging tests for abscess detection around the native valves, TTE detected none, TEE detected 4 (out of 11), and CCT detected all 12. Additionally, TTE + TEE + CCT detected all 11. The sensitivity was TTE 0% and TEE 36.4%, whereas CCT and mutual use of imaging tests had the same sensitivity of 100%.

Around the prosthetic valves, in single-test application for the detection of abscesses, TTE identified 3 out of 8, TEE 4 out of 8, and CCT 7 out of 8, the same as TTE + TEE + CCT. The sensitivity and specificity were TTE 42.9%/100%, TEE 57.1%/100%, and CCT 100%/0%, the same as for mutual application, 100%/0%.

In CCT examinations, the mean depth of abscesses was 13.32 ± 10.43 mm (95% Cl 7.54–19.10), with minimal to maximal values at 2.5 and 39 mm. The mean width of abscesses was 26.79 ± 16.65 mm (95% Cl 16.21–37.37), with minimal value 6.9 mm and maximum value 52.1 mm.

Performance of diagnostic tests is summarized in Table 6.

### 3.7. Pseudoaneurysms

In detection of pseudoaneurysms around all valve types, TTE identified 4 (out of 46) and TEE 17 (out of 41), whereas CCT identified 44 (out of 46) and TTE + TEE + CCT 39 (out of 40). The calculated diagnostic performance for sensitivity and specificity of imaging tests used individually were TTE 9.1%/100%, TEE 42.5%/100%, and CCT 100%/0%. In mutual usage of imaging tests, the sensitivity and specificity were the same as in CCT used alone. Around the native valves, the sensitivity and specificity in pseudoaneurysm detection in single-test application were low: TTE 4%/100% and TEE 26.1%/100%. CCT used alone and the mutual application of all three tests, however, had the sensitivity of 100% and specificity of 0%. Around the prosthetic valves, TTE identified 3 pseudoaneurysms out of 19 and TEE 11 out of 17, whereas CCT detected all 19, and TTE + TEE + CCT identified all 17. The sensitivity in pseudoaneurysm detection was TTE 15.8%, TEE 64.7%, and CCT 100%. If we used imaging tests together, the sensitivity was also 100%.

We tested diagnostic performance of echocardiography and cardiac CT in relation to localization around the valves, which were divided into two categories. One category collected pseudoaneurysms anterior and to the right side of the valve. The other category collected pseudoaneurysm posterior, to the left side of the valve, and in intervalvular fibrosa. In anterior right-sided localization, TTE did not detect any pseudoaneurysms; TEE identified 3 out of 13, but CCT detected 15 out of 16 and TTE + TEE + CCT detected all 12. The sensitivity was TTE 0% and TEE 23.1%, whereas for CCT and mutual-used tests 100%. In posterior left-sided intervalvular fibrosa localization, total number of detected pseudoaneurysms by TTE was 3 (out of 22), TEE 12 (out of 20), CCT 21 (out of 22), and TTE + TEE + CCT 19 (out of 20). The calculated sensitivity was TTE 14.3%, TEE 63.2%, and CCT 100%. In the same localization, when the imaging tests were applied together, the sensitivity was the same as single-applied CCT.

In CCT examinations, the mean depth of abscesses was 10.33 ± 6.46 mm (95% Cl 8.39–12.27), with minimal to maximal values at 3.1 and 31.5 mm. The mean width of abscesses was 18.76 ± 10.98 mm (95% Cl 15.50–22.2), with minimal value 5.0 mm and maximum value 48.4 mm.

Performance of diagnostic tests is summarized in Table 7 and Table 8.

### 3.8. Fistulas

Owing to a small sample, only three fistulas were detected; we did not test the diagnostic performance of echocardiography and cardiac CT. Out of the three fistulas, TTE and TEE detected two whereas CCT detected all three. The mean width of fistulas determined using CCT examination was 8.6 ± 2.17 mm (95% Cl 3.20–13.99), with the minimal and maximum value at 6.1 mm and 10.0 mm.

### 3.9. Leaks

Owing to a small sample, only two leaks were found; we did not test the diagnostic performance of echocardiography and cardiac CT. TTE missed one leak, whereas TEE and CCT detected both.

In summary, in testing the diagnostic performance of CCT, TTE, and TEE, higher sensitivity is obtained when the three tests are applied together. As proposed, at least one is positive compared to individual application.

### 3.10. Image Quality Improvement

A total of 78 examinations were examined, out of which row data were reconstructed with FBP in 40 examinations (51.3%) and with iterative reconstructions in 38 examinations (48.7%). There were 34 examinations with satisfactory image quality (43.6%) and 44 examinations with excellent image quality (56.4%). In the group with FBP reconstruction, there were 13 examinations with satisfactory image quality (38.2%) and 27 with excellent image quality (61.4%). In the group with iterative reconstruction, there were 21 examinations with satisfactory quality (61.8%) and 17 examinations with excellent quality (38.6%). Filtered-back projection is significantly more common with excellent image quality (*p* = 0.043).

In the 34 examinations with satisfactory image quality, the mean voltage was 111.18 ± 13.203 kV, and in the 44 examinations with excellent image quality, mean voltage was 117.27 ± 9.242 kV. Tube voltage was significantly higher in the excellent image quality group compared to the satisfactory image quality group (t = 2.397; *p* = 0.026).

## 4. Discussion

In this study, CCT was superior to TTE and TEE in detection of valvular and paravalvular IE lesions, which was surprisingly different from previous studies concerning visualization of valvular IE lesions. In previous studies, in NVE, the diagnostic sensitivity and specificity for TTE is 50–90% and 90%, respectively, whereas TEE is >90% for 2D TEE. In previous studies, in PVE, the sensitivity of TTE and TEE was 40–70% and 85%, respectively [17]. In comparison to the study of Sifaoui et al., who compared sensitivity and specificity of all IE lesions, valvular and paravalvular for single usage of TEE and CCT, and for TEE use against mutual usage TEE and CCT, our results were similar in mutual usage, but surprisingly low in single usage of TEE. They found sensitivity of TEE for valvular and paravalvular lesions at 93.6%, as opposed to ours at 58.7%, and for valvular lesions 98.2% and paravalvular lesions 79.5%, which are contrary to ours at 65.5% and 46% [20]. Explaining the unexpected low TEE sensitivity, a significant number of patients were complex with corrected/uncorrected CHD, BAV, or heavily calcified valvula near the annulus, often with more than one affected valvula with several different valvular and paravalvular IE manifestations, not all of which were seen by TEE, but were seen by CCT, due to better spatial resolution, which was confirmed by surgery or autopsy. We calculated diagnostic performance of all lesion types and the specific IE lesion, in both native and prosthetic valves in all four potential localizations and in pacemaker leads and shunts, which probably lowered the sensitivity of TEE. Similar to our results, Sifaoui et al. found high sensitivity of CCT (96.8%) in the detection of valvular and paravalvular lesions, and for mutual usage of TEE+CCT in detection of valvular lesions (98.2%) and paravalvular lesions (91.6%), arriving at a similar conclusion, that mutual usage raises the diagnostic performance of both tests [20].

**Figure 1:**
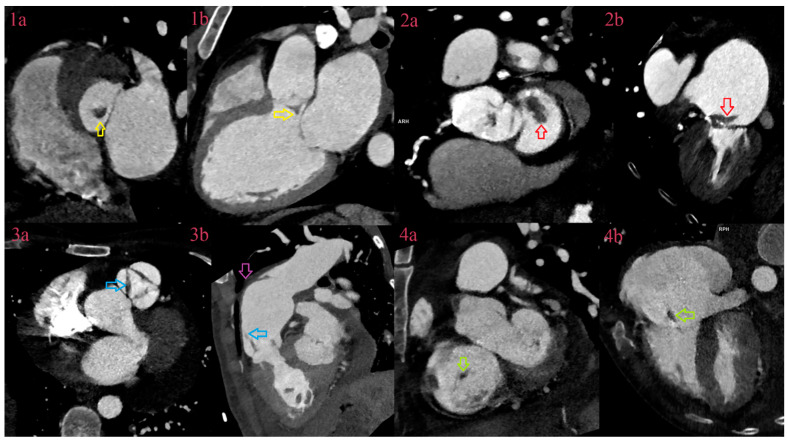
CCT of vegetations in cardiac valves. CCT depicts vegetation of the aortic valve attached to the aneurysm of noncoronary cusps (yellow arrow) in the LVOT plane (**1a**) and 3-chamber view (**1b**). CCT depicts vegetation of the mitral valve (red arrow) attached to the A1 segment of anterior cusps in the mitral plane (**2a**) and 3-chamber view (**2b**). CCT depicts thickening in all three cusps of the pulmonary valve (blue arrow) (**3a**) with vegetation attached to the right cusps in 3-chamber view (blue arrow) (**3b**). Additionally, image 3 depicts abscess in the anterior wall of the pulmonal artery with vegetation (purple arrow). CCT depicts vegetation (green arrow) of the tricuspid valve attached to the anterior cusps in the valve plane (**4a**) and 3-chamber view (**4b**).

In analysis per specific IE lesion, diagnostic performance of CCT in detection of vegetations in natives and prosthetic valves was similar to the previous studies, with sensitivity between 70 and 96% and specificity between 20 and 100% (Figure 1) [20,21,22,23,24,25,26,27].

**Figure 2:**
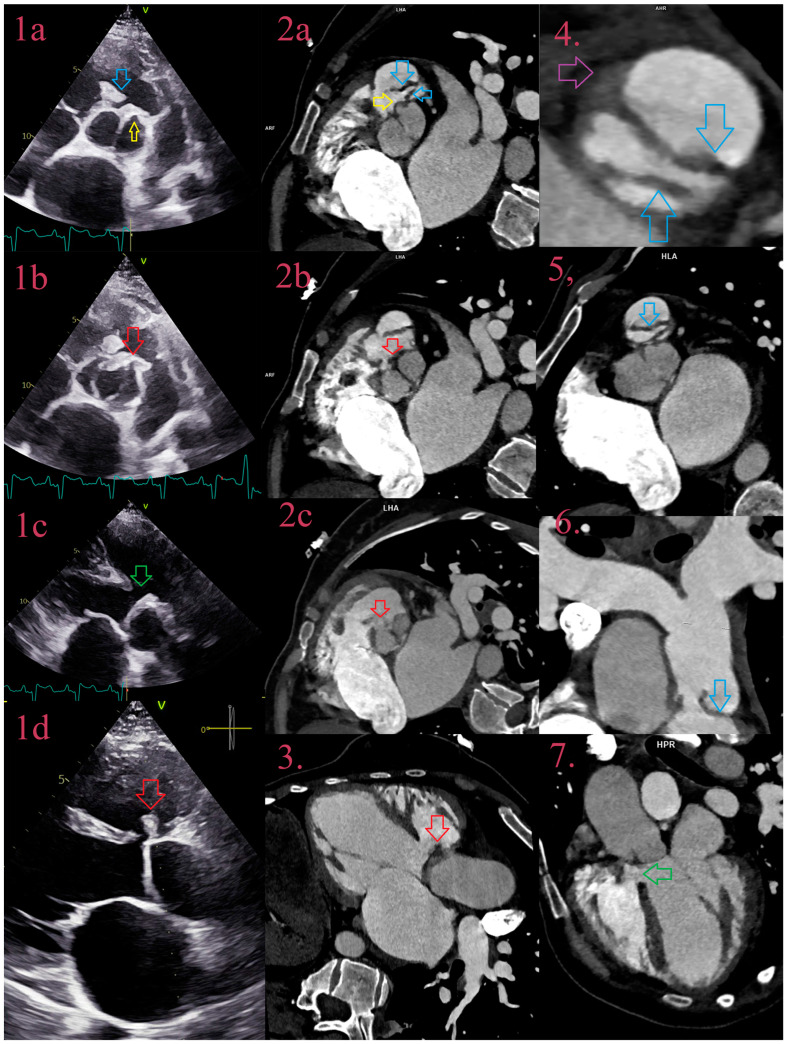
IE in an uncorrected congenital heart defect—Tetralogy of Fallot. Comparative overview of TEE and CCT: TEE in the aortic plane depicts IE of 2-sinus bicuspid aortic valve (BAV), latero-lateral phenotype, with small aneurysm of “left” cusps near anterior commissure (yellow arrow) in closed cusps (**1a**) and attached vegetation (red arrow) in open cusps (**1b**), with a thickened leaflet of the pulmonary valve due to IE (blue arrow) (**1a**). TEE in 3-chamber view depicts ventricular septal defect (green arrow) (**1c**) and prolapse of vegetation, attached to the left cusp (red arrow), in VSD (**1d**). CCT, in aortic plane, depicts IE of BAV and the pulmonary valve, with aneurysm (yellow arrow) and vegetation (red arrow) of left aortic cusps (**2a**–**2c**), and two thickened asymmetric pulmonary cusps (blue arrow) (**2a**). CCT in 3-chamber view depicts VSD (green arrow) with prolapse of vegetation (red arrow) from left cusps of BAV (**3**,**7**). CCT depicts an asymmetric thickened bicuspid pulmonary valve (blue arrow) with small abscess (purple arrow), in the plane of the pulmonary valve (**4**,**5**) and in coronal reformation (**6**).

As expected, the diagnostic performance of TTE was similar to the previous studies, but for TEE it was surprisingly lower [17]. In explanation, TEE was performed in 20 fewer patients, compared to TTE and CCT, with a smaller number of vegetations to be detected (48 out of 65). Missed vegetations were usually attached to heavily calcified valves, sometimes too small to be detected, or in cases of severe destruction, as in the bicuspid aortic valve, firmly attached, partially calcified, without oscillating movements, or sometimes hidden in commissural part, adjacent to the wall of LVOT. In a few cases, parts of large perforated aneurysms were described as vegetations. Additionally, missed vegetations were attached to the prosthetic valve, especially in anterior localization, or when there was more than one vegetation. CCT showed superior visualization of vegetations due to better spatial resolution, especially in both right- and left-sided NVE (Figure 2), but it was inferior in detecting vegetation around pacemaker leads, detecting 2 out of 4, and around some prosthetic valves, due to strike artifacts of metal origin and small vegetation size.

To our knowledge, there have not been studies to compare the detection of aneurysms of valve leaflets between CCT, TTE, and TEE, in single and mutual usage to the operative findings. CCT was superior to TTE and TEE in detection, due to better spatial resolution, with sensitivity 100%, but low specificity, 10.5%, probably as some small-sized aneurysms were not detected in surgery. In its mutual usage, CCT was the main diagnostic test.

**Figure 3:**
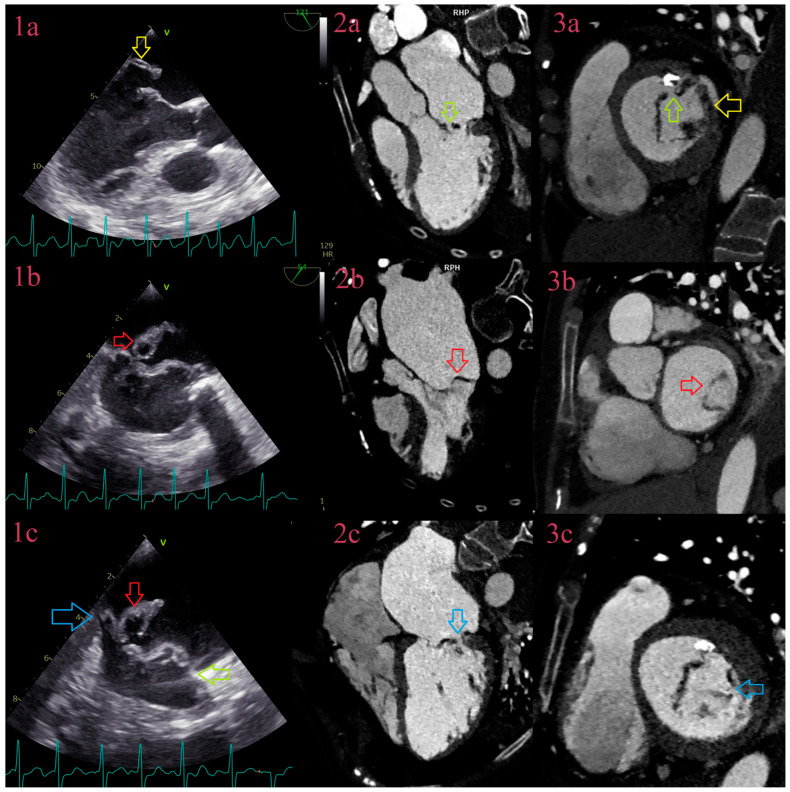
IE of the mitral valve on the field of posterior cusps prolapse with vegetations and perforations. Comparative overview of TEE and CCT in IE of the mitral valve: TEE in 3-chamber view depicts thickened posterior cusps (yellow arrow) (**1a**), with aneurysm of the P2 segment of posterior cusps (red arrow), on the field of previous prolapse, (**1b**), and perforation near attachment of the P2 segment (blue arrow) (**1c**). CCT depicts thickened anterior cusps with perforation of the A2 segment (green arrow), in 3-chamber view (**2a**) and 2-chamber view and thickened posterior cups (yellow arrow) (**3a**). A2 perforation was additionally confirmed in TEE (green arrow) (**1c**). CCT depicts aneurysm of the P2 segment of posterior cusps in 3-chamber view (red arrow) (**2b**) and 2-chamber view (**3b**). CCT confirmed perforation of the P2 segment of posterior cusps (blue arrow) in 3-chamber view (**2c**) and 2-chamber view (**3c**).

**Figure 4:**
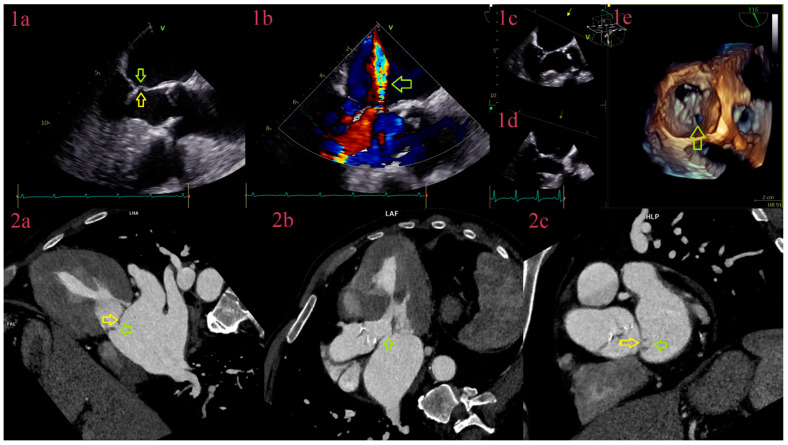
Perforation of anterior cusp of the mitral valve. Comparative overview of TEE and CCT in IE of the mitral valve. TEE, in 3-chamber view, depicts thickened aneurysm of anterior cusps (yellow arrow) with perforation (green arrow) (**1a**), confirmed with color Doppler flow with two-jet phenomenon (**1b**) and in volume-rendering reformation (**1c**–**1e**). CCT, in 3-chamber view, depicts aneurysm of anterior cusps (yellow arrow) with perforation at its top (green arrow) (**2a**,**2b**) and in the plane of the mitral valve (**2c**).

In our study, detection of perforations by CCT was superior to TTE and TEE (Figure 3), with higher sensitivity and specificity in comparison to the previous studies, where for CCT was in a range between 41–68.4% and 73.5–96.3%, and for TEE 68.4–81.3% and 89.8–96% [20,25,27]. We have also found that mutual usage of diagnostic tests raises the sensitivity to 88.9%, with CCT as the main diagnostic test, in a comparison to single usage, where sensitivity for TTE was 42.9%, TEE 63.9%, and CCT 83.7%. The main advantage of echocardiography to cardiac CT was utilization of color Doppler effect and detection of two-jet phenomenon in a real time as a sign of perforation (Figure 4), especially in small-sized perforations in heavily calcified valvula. If there were more than one perforation, however, usually in left-sided IE, in nearby aortic cusps or in mitral valve, at the same leaflet, in nearby segments, or near the annulus, not all were detected by TEE. Technology advancement in CCT with utilization of double-oblique reformations has improved spatial resolution and increased detection rates.

In detection of abscesses, the CCT was the superior imaging method even as a single test in comparison to TTE and TEE, between which the performance of TTE was extremely low. Sensitivity of TTE in abscess detection around all valves was 15.8%, whereas it missed all abscesses around native valves. Sensitivity of TEE was higher, 44.4%, in all valve types, but lower around the native valves, 36.4%, and higher around the prosthetic valves, 57.1%. Contradictorily, we expected poor visualization around prosthetic valves due to metal artifacts but were surpassed by poor visibility around native valves, especially where part of the abscess did not drain. A probable explanation lies in the echogenicity of the abscess, which is brighter and similar to the echogenicity of surrounding epicardial fat tissue, especially in the early stages of formation. Also, in the early postoperative period, hematomas and edema led to wrong conclusions and false positive results in TEE examination. On the contrary, CCT depicted all 20 abscesses independent of the valve type and localization. It was especially superior in detecting a patch abscess after repair of the ventricular septal defect. It was difficult to compare our findings with previous studies where abscesses and pseudoaneurysms were observed as one category. Sifaoui et al. divided abscesses and pseudoaneurysms into two precise categories, where sensitivity and specificity of TEE was higher and that of CCT lower compared to our results. Sensitivity and specificity of TEE for both valve types were 72.7%/89.1%, the native valves 73.3%/92.1%, and the prosthetic valves 71.4%/75%, whereas for CCT for all valves were 77.3%/80.4%, the native valves 80%/86.8%, and the prosthetic valves 71.4%/50% [20]. CCT is an excellent imaging tool in abscess visualization independent of localization, but its detection poses cautious inspection, especially in small-sized abscesses, as its density is similar to the density of surrounding fat, but still visibly brighter. To minimize false positive results when reading a potential graft infection, finding circumferential thin hypodensity <3 mm is considered normal because of graft material.

**Figure 5:**
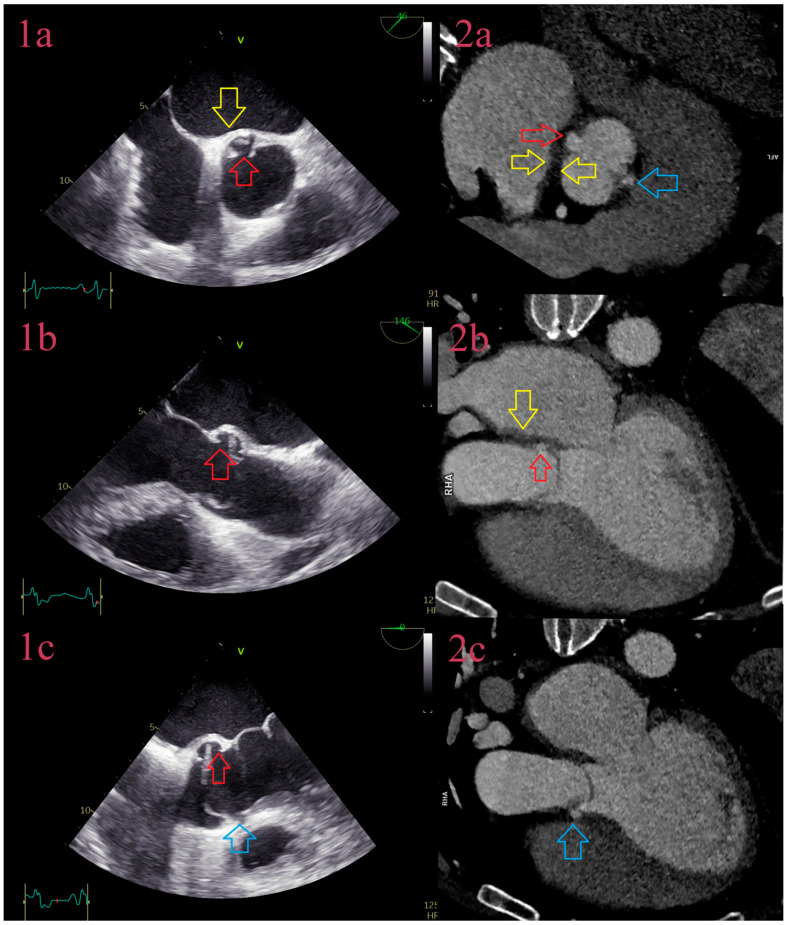
IE of the bicuspid aortic valve (BAV). Comparative overview of TEE and CCT, in the patient with IE of 2-sinus BAV, latero-lateral phenotype, depicting development of abscesses and pseudoaneurysms in commissural parts of both cusps. TEE depicts hyperechogenic-thickened intervalvular fibrosa as a sign of abscess (yellow arrow) and an anechoic cavity in posterior commissure as a sign of pseudoaneurysm (red arrow) (**1a**). CCT, at aortic plane, depicts contrast-filled cavity in anterior (blue arrow) and posterior commissure (red arrow) of BAV cusps as a sign of pseudoaneurysms, and hypoechogenic abscess of intervalvular fibrosa (yellow arrow) (**2a**). (**1b**,**2b**) in 3-chamber view depicts posterior pseudoaneurysm (red arrow). (**1c**,**2c**) in 3-chamber view, depicts anterior pseudoaneurysms (blue arrow).

**Figure 6:**
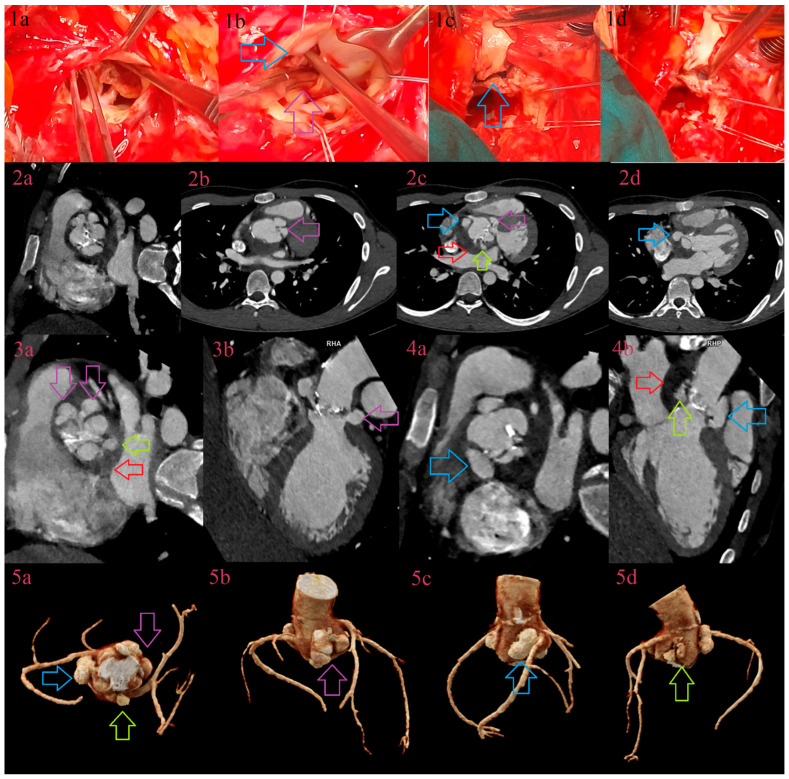
IE in corrected bicuspid aortic valve due to Ozaki procedure. Comparative overview of surgical findings and CCT: a degenerative corrected aortic valve in a 19-year-old male patient is demonstrated using surgery instruments (**1a**), entering in paravalvular cavities between the right coronary cusp (RCC) and left coronary cusp (LCC) (purple arrow), and between RCC and noncoronary cusp (NCC) (blue arrow) (**1b**). The instruments also reveal the depth of the largest pseudoaneurysm between RCC and NCC, which abuts right coronary sinus (**1c**,**1d**). CCT depicts the degenerative aortic valve in the aortic plane (**2a**). CCT depicts two pseudoaneurysms between RCC and LCC (purple arrows) in axial plane (**2b**), aortic plane (**3a**), 3-chamber plane (**3b**), and volume rendering (**5a**,**5b**). CCT depicts the largest pseudoaneurysm between RCC and NCC (blue arrows) in axial plane (**2c**,**2d**), aortic plane (**4a**), 3-chamber plane (**4b**), and volume rendering (**5a**,**5c**). The smallest pseudoaneurysm between LCC and NCC (green arrow) surrounded by abscess of intervalvular fibrosa (red arrow) depicted in axial plane (**2c**), aortic plane (**3a**), 3-chamber plane (**4b**), and volume rendering (**5a**,**5d**).

Similar to the detection of abscesses, CCT was superior in detecting pseudoaneurysms in comparison to TTE and TEE, even as a single-applied imaging test (Figure 5). In comparison to other authors, diagnostic performance of TEE was similar to the lower values, whereas CCT was similar or higher. In comparable studies, sensitivity and specificity of TEE was within the range 40–100% and 66.7–100%, and for CCT 60–100% and 75–100% [20,21,22,23,24,25,26,27]. Diagnostic performance of TTE was extremely low, as in detection of abscesses, whereas TEE was lower than expected in comparison to CCT. A reasonable explanation for the discrepancy in sensitivity and specificity between TEE and CCT (sensitivity TTE vs. CCT for all valves 42.5/100% vs. 100/0%, for NVE 26.1/100% vs. 100/0%, and for PVE 64.7%/NR vs. 100/0%) would be that echocardiography detected one pseudoaneurysm, whereas there were more than one, and CCT saw them all (Figure 6). Also, in mutual usage CCT with TTE and TEE, sensitivity was 100%, and CCT was the main diagnostic imaging test. Habets et al. presented similar findings in PVE, where the sensitivity and specificity of TTE+CCT were 68/91% (in our study 64.7%/NR), but when CCT was added, sensitivity and specificity rose to 100/91% (in our study 100/0%) [28]. Also, in testing diagnostic performance dependent on localization, we detected that it was low in TTE independent of localization, whereas TEE showed better sensitivity in the posterior and left-sided zone around the valve and in intervalvular fibrosa, in comparison to the anterior and right-sided zone around the valve (sensitivity and specificity 63.2/100% vs. 25%/NR). CCT in single and mutual application depicted all pseudoaneurysms due to better spatial resolution.

**Figure 7:**
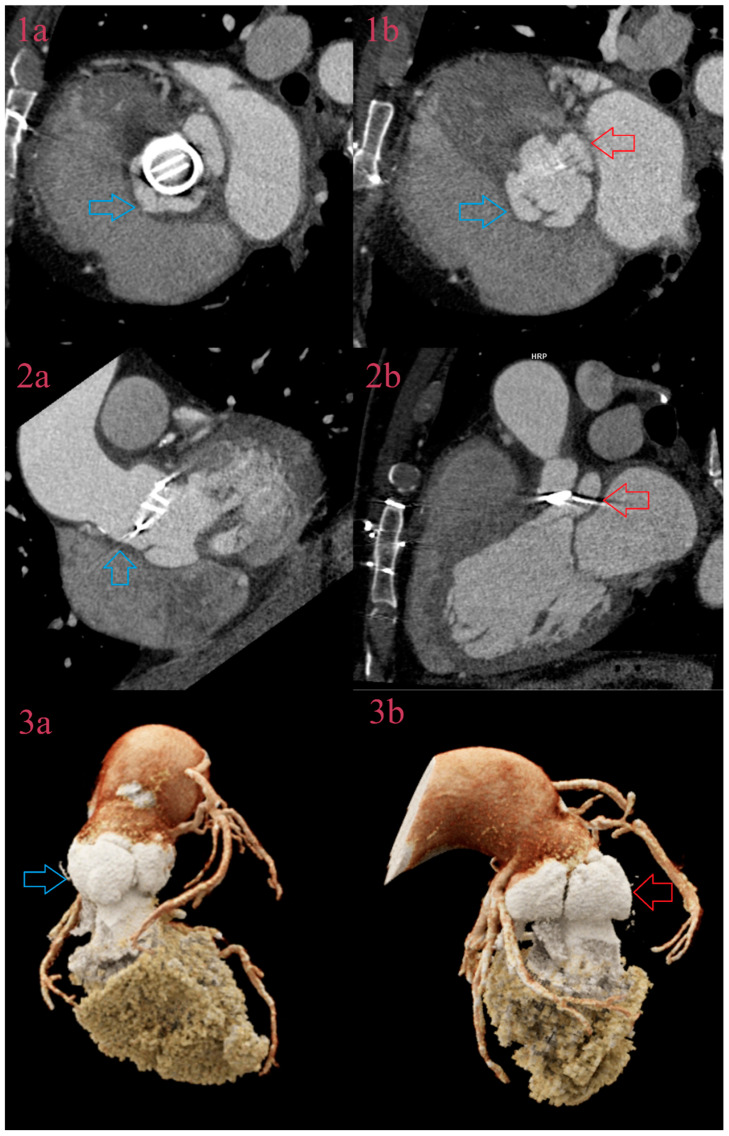
Fistula and pseudoaneurysm around a prosthetic aortic valvula. CCT of IE of the prosthetic aortic valvula, in the aortic plane, depicts development of two contrast-filled cavities, to the left, the atrium (red arrow), and to the right, the outflow tract (blue arrow) at the level of prosthesis (**1a**), and at the level of the left ventricle outflow tract (LVOT) (**1b**). CCT in 3-chamber view depicts communication of the anterior contrast-filled cavity with the left ventricle (blue arrow) as a sign of fistula (**2a**). CCT in 3-chamber view depicts a posterior contrast-filled cavity with a posterior wall of LVOT as a sign of pseudoaneurysm (red arrow) (**2b**). Volume rendering reconstruction depicts the fistula (**3a**) and pseudoaneurysm (**3b**).

As we found a very small number of leaks and fistulas, we do not provide diagnostic performance in this report due to small sample size, but CCT was the superior imaging method in both single and mutual use (Figure 7). In detection of leaks, the reader should have in mind not to mistake sewing a ring around the leaking prosthetic valve, as the leak is small a contrast-filled lesion but asymmetric and larger compared to the normal postsurgical finding.

Precision in detecting paravalvular and valvular lesions, especially, depends greatly on the image quality. In our study, we found that image quality is statistically significant, better in a group with FBP reconstruction, where we did not use dose modulation protocol, to the group with iterative reconstruction protocol, where we used dose modulation protocol. As the normal thickness of the valve leaflet is one millimeter, it is advisable not to reduce tube voltage by the dose modulation protocols, as higher kV values are in statistically significant correlation with excellent image quality. Although higher tube voltages increase radiation exposure to the patient, a precise answer to the existing and often life-threatening clinical problem overrates risks of radiation exposure. We have not found similar studies to ours in order to compare it.

## 5. Limitations of the Study

One limitation in this study was that the lesion groups were small. We investigated the diagnostic performance of CCT, which exposed patients to the ionizing radiation. Another limitation was the inability to detect the true negative group of patients, which resulted in inability to calculate real specificity. A further limitation was that during the 10-year time span of patient selection, we could not perform echocardiography by the same operator, and there was a time lag of more than two weeks between surgery and the echocardiography and cardiac CT, which resulted in discrepancies in diagnostic performance.

## 6. Conclusions

In the anatomical assessment of infective endocarditis, cardiac computed tomography has emerged as a powerful imaging method. Lacking hemodynamic assessment, CCT can be classified as an additional imaging method in the regular clinical routine for patients who suffer from infective endocarditis.

## Figures and Tables

**Table 1:** Demographic and clinical characteristics of the population.

Variable	Mean ± SD or Median and IQR	Minimum–Maximum Number/ Percentage
Age	52.29 ± 16.62	14–84
Male to Female	56:22	71.8%:28.2%
Heart Rate (bpm)	78.00 ± 13.75	40–117
Ejection fraction (%)	56 ± 13	20–81
Risk factors		
Degenerative valve disease	10	12.8%
Prosthetic valve	13	16.7%
Bicuspid aortic valve	14	17.9%
Mitral valve prolapse	7	9.0%
Uncorrected congenital heart defect	3	3.8%
Corrected congenital heart defect	5	6.4%
Pacemaker leads	4	5.1%
Positive blood culture	47 out of 74 (4 missing)	60.3%
Causative microorganisms		
Staphylococcus species		23.2%
Streptococcus species		10.4%
Enterococcus species		16.7%
Others		11.7%
Laboratory findings		
White blood cell counts [10^9^/L)	11.00 ± 7.79	3.2–62.4
C-reactive protein (mL/L)	86.30 ± 82.17	3.2–335.6
Fibrinogen (g/L)	5.33 ± 1.93	2.1–12.0
Procalcitonin (ng/mL)	0.15 ± 3.19	0.03–14.61
Creatinine (µmol/L)	89.50 ± 199.66	53–1121
Systemic emboli in specific organ		
Absent	51	65.4%
Brain	3	3.8%
Lungs	4	5.1%
Liver	2	2.6%
Spleen	11	14.1%
Kidney	1	1.3%
Muscles	1	1.3%
Vessels	1	1.3%
Spondylodiscitis	2	2.6%
Coronary artery disease		
Absent	42	53.8%
Intermediate stenosis	22	28.2%
Significant stenosis	8	10.3%
Mycotic pseudoaneurysms	6	7.7%

**Table 2:** Diagnostic performance of echocardiography and cardiac CT in the detection of valvular and paravalvular lesions.

Diagnostic Performance	TTE	TEE	CCT	TTE + TEE + CCT
**Valvular and paravalvular lesions**				
**Accuracy**	50.6%	59%	85.7%	87.9%
**95% Cl**	44.4–56.8	52.8–66.4	81.3–90.0	83.3–92.4
**Sensitivity**	46.2%	58.7%	94.2%	97.8%
**95% Cl**	39.6–52.7	51.4–65.9	91.1–97.2	95.6–99.9
**Specificity**	85.7%	68.4%	17.9%	0%
**95% Cl**	72.8–98.7	47.5–89.3	3.7–32.0	NR
**PPV**	96.3%	94.6%	90.1%	89.7%
**NPV**	16.7%	14.6%	27.8%	NR
**Valvular lesions**				
**Accuracy**	63.3%	65.4%	81.7%	83.6%
**95% Cl**	56.3–70.4	57.3–73.5	76.0–87.3	77.3–89.9
**Sensitivity**	60%	65.5%	91.6%	96.6%
**95% Cl**	52.3–67.7	56.9–74.2	87.2–96.0	93.2–99.9
**Specificity**	84%	57.9%	20%	0%
**95% Cl**	69.6–98.4	35.2–80.6	4.3–35.7	NR
**PPV**	95.9%	92.7%	87.7%	86.2%
**NPV**	25.3%	21.6%	27.8%	NR
**Paravalvular lesions**				
**Accuracy**	18.3%	47.7%	95.8%	96.9%
**95% Cl**	9.9–27.3	35.5–59.8	91.1–100.5	92.6–101.1
**Sensitivity**	14.7%	46%	100%	100%
**95% Cl**	6.3–23.1	33.7–58.3	100–100	100–100
**Specificity**	100%	10.5%	0%	0%
**95% Cl**	100–100	10.5–10.5	NR	NR
**PPV**	100%	100%	95.8%	96.9%
**NPV**	4.9%	5.6%	NR	NR

Cl—Confidence level, PPV—Positive predictive value, NPV—Negative predictive value, NR—No results.

**Table 3:** Diagnostic performance of echocardiography and cardiac CT in the detection of vegetations.

Vegetation	TTE	TEE	CCT	TTE + TEE + CCT
**All valves**				
**Accuracy**	80.7%	75.4%	92%	92.4%
**95% Cl**	72.4–88.9	64.9–85.9	86.4–97.7	86.0–98.8
**Sensitivity**	84.3%	78.7%	94%	100%
**95% Cl**	76.5–92.2	68.4–89.0	88.9–99.1	100–100
**Specificity**	20%	25%	60%	0%
**95% Cl**	0–55.1	0–67.4	17.1–102.9	NR
**PPV**	94.6%	94%	97.5%	92.4%
**NPV**	7.1%	6.7%	37.5%	NR
**Native valves**				
**Accuracy**	82.4%	77.8%	95.9%	90.9%
**95% Cl**	73.8–91.1	66.7–88.9	91.5–100.4	83.3–98.5
**Sensitivity**	87%	82%	98.6%	100%
**95% Cl**	79.0–94.9	71.4–92.6	95.7–101.4	100–100
**Specificity**	20%	25%	60%	0%
**95% Cl**	0–55.1	0–67.4	17.1–102.9	NR
**PPV**	93.8%	93%	97.1%	90.9%
**NPV**	100%	9.1%	75%	NR
**Prosthetic valves**				
**Accuracy**	71.4%	63.6%	71.4%	100%
**95% Cl**	47.8–95.1	35.2–92.1	47.8–95.1	100–100
**Sensitivity**	71.4%	63.6%	71.4%	100%
**95% Cl**	47.8–95.1	35.2–92.1	47.8–95.1	100–100
**Specificity**	NR	NR	NR	NR
**95% Cl**				
**PPV**	100%	100%	100%	100%
**NPV**	NR	NR	NR	NR

Cl—Confidence level, PPV—Positive predictive value, NPV—Negative predictive value, NR—No results.

**Table 4:** Diagnostic performance of echocardiography and cardiac CT in the detection of aneurysms.

Aneurysms	TTE	TEE	CCT	TTE + TEE + CCT
**Native valves**				
**Accuracy**	50%	48.4%	59.5%	61.3%
**95% Cl**	34.9–65.1	30.8–66.0	44.7–74.4	44.1–78.4
**Sensitivity**	8.7%	31.6%	100%	100%
**95% Cl**	0–20.2	10.7–52.5	100–100	100–100
**Specificity**	100%	75%	10.5%	0%
**95% Cl**	100–100	50.5–99.5	0–24.3	NR
**PPV**	100%	66.7%	57.5%	61.9%
**NPV**	47.5%	40.9%	100%	NR

Cl—Confidence level, PPV—Positive predictive value, NPV—Negative predictive value, NR—No results.

**Table 5:** Diagnostic performance of echocardiography and cardiac CT in the detection of perforations.

Perforation	TTE	TEE	CCT	TTE + TEE + CCT
**Native valves**				
**Accuracy**	44%	64.9%	82%	86.5%
**95% Cl**	30.2–57.8	49.5–80.2	71.4–92.6	75.5–97.5
**Sensitivity**	42.9%	63.9%	83.7%	88.9%
**95% Cl**	29–56.7	48.2–79.6	73.3–94.0	78.6–99.2
**Specificity**	100%	100%	0%	0%
**95% Cl**	100–100	100–100	NR	NR
**PPV**	100%	100%	97.6%	97%
**NPV**	3.4%	7.1%	NR	NR

Cl—Confidence level, PPV—Positive predictive value, NPV—Negative predictive value, NR—No results.

**Table 6:** Diagnostic performance of echocardiography and cardiac CT in the detection of abscesses.

Abscesses	TTE	TEE	CCT	TTE + TEE + CCT
**All valves**				
**Accuracy**	20%	47.4%	95%	94.7%
**95% Cl**	2.5–37.5	24.9–69.8	85.4–104.6	84.7–104.8
**Sensitivity**	15.8%	44.4%	100%	100%
**95% Cl**	0–32.2	21.5–67.4	100–100	100–100
**Specificity**	100%	100%	0%	0%
**95% Cl**	100–100	100–100	NR	NR
**PPV**	100%	100%	95%	94.7%
**NPV**	5.9%	9.1%	NR	NR
**Native valves**				
**Accuracy**	0%	36.4%	100%	100%
**95% Cl**	0–0	7.9–64.8	100–100	100–100
**Sensitivity**	0%	36.4%	100%	100%
**95% Cl**	NR	7.9–64.8	100–100	100–100
**Specificity**	NR	NR	NR	NR
**95% Cl**				
**PPV**	NR	100%	100%	100%
**NPV**	NR	NR	NR	NR
**Prosthetic valves**				
**Accuracy**	50%	62.5%	87.5%	87.5%
**95% Cl**	15.4–84.6	29.0–96.0	64.6–110.4	64.6–110.4
**Sensitivity**	42.9%	57.1%	100%	100%
**95% Cl**	6.2–79.5	20.5–93.8	100–100	100–100
**Specificity**	100%	100%	0%	0%
**95% Cl**	100–100	100–100	NR	NR
**PPV**	100%	100%	87.5%	87.5%
**NPV**	20%	25%	NR	NR

Cl—Confidence level, PPV—Positive predictive value, NPV—Negative predictive value, NR—No results.

**Table 7:** Diagnostic performance of echocardiography and cardiac CT in the detection of pseudoaneurysms.

Pseudoaneurysms	TTE	TEE	CCT	TTE + TEE + CCT
**All valves**				
**Accuracy**	13%	43.9%	95.7%	97.5%
**95% Cl**	3.3–22.8	28.7–59.1	89.8–101.5	92.7–102.3
**Sensitivity**	9.1%	42.5%	100%	100%
**95% Cl**	0.6–17.6	7.2–57.8	100–100	100–100
**Specificity**	100%	100%	0%	0%
**95% Cl**	100–100	100–100	NR	NR
**PPV**	100%	100%	95.7%	97.5%
**NPV**	4.8%	4.2%	NR	NR
**Native valves**				
**Accuracy**	11.1%	29.2%	92.6%	95.7%
**95% Cl**	0–23.0	11.0–47.4	82.7–102.5	87.3–104.0
**Sensitivity**	4%	26.1%	100%	100%
**95% Cl**	0–11.7	8.1–44.0	100–100	100–100
**Specificity**	100	100%	0%	0%
**95% Cl**	100–100	100–100	NR	NR
**PPV**	100%	100%	92.6%	95.7%
**NPV**	7.7%	5.6%	NR	NR
**Prosthetic valves**				
**Accuracy**	15.8%	64.7%	100%	100%
**95% Cl**	0–32.2	42.0–87.4	100	100–100
**Sensitivity**	15.8%	64.7%	100%	100%
**95% Cl**	0–32.2	42.0–87.4	100–100	100–100
**Specificity**	NR	NR	0	NR
**95% Cl**			NR	
**PPV**	100%	100%	100%	100%
**NPV**	0%	0%	NR	NR

Cl—Confidence level, PPV—Positive predictive value, NPV—Negative predictive value, NR—No results.

**Table 8:** Diagnostic performance of echocardiography and cardiac CT in pseudoaneurysm detection according to localization.

Localization	TTE	TEE	CCT	TTE + TEE + CCT
**Pseudoaneurysms—Posterior and to the left side of the valve and in intervalvular fibrosa**				
**Accuracy**	18.2%	62.5%	95.5%	95%
**95% Cl**	2.1–34.3	44.1–85.9	86.8–104.2	85.4–104.6
**Sensitivity**	14.3%	63.2%	100%	100%
**95% Cl**	0–29.3	41.5–84.8	100–100	100–100
**Specificity**	100%	100%	0%	0%
**95% Cl**	100–100	100–100	NR	NR
**PPV**	100%	100%	95.5%	95%
**NPV**	5.3%	12.5%	NR	NR
**Pseudoaneurysms—Anterior and to the right side of the valve**				
**Accuracy**	6.3%	23.1%	93.8%	100%
**95% Cl**	0–18.1	0.2–46.0	81.9–105.6	100–100
**Sensitivity**	0%	23.1%	100%	100%
**95% Cl**	NR	0.2–46.0	100–100	100–100
**Specificity**	100%	NR	0%	NR
**95% Cl**	100–100		NR	
**PPV**	NR	100%	93.8%	100%
**NPV**	6.3%	NR	NR	NR

Cl—Confidence level, PPV—Positive predictive value, NPV—Negative predictive value, NR—No results.

## Data Availability

The data presented in this study are available upon reasonable request from the first and the corresponding author.
